# CCDC69 is a prognostic marker of breast cancer and correlates with tumor immune cell infiltration

**DOI:** 10.3389/fsurg.2022.879921

**Published:** 2022-07-15

**Authors:** Yi Yi, Tao Xu, Yufang Tan, Wenchang Lv, Chongru Zhao, Min Wu, Yiping Wu, Qi Zhang

**Affiliations:** ^1^ Department of Plastic and Cosmetic Surgery, Tongji Hospital, Tongji Medical College, Huazhong University of Science and Technology, Wuhan, China; ^2^ Department of Thyroid and Breast Surgery, Tongji Hospital, Tongji Medical College, Huazhong University of Science and Technology, Wuhan, China

**Keywords:** CCDC69, breast cancer, prognosis, immune infiltration, PD-1/PD-L1

## Abstract

**Purpose:**

Breast cancer (BC) is the most common malignancy and the leading cause of cancer-related death among women worldwide. Early detection, treatment, and metastasis monitoring are very important for the prognosis of BC patients. Therefore, effective biomarkers need to be explored to help monitor the prognosis of BC patients and guide treatment decisions.

**Methods:**

In this study, the relationship between CCDC69 expression levels and tumor clinical characteristics were analyzed using RNA-seq information in BC samples from the TCGA database. Kaplan-Meier survival analysis was performed to analyze the prognostic value of CCDC69 in BC patients. Besides, gene enrichment analysis in BC samples was used to confirm the main function of CCDC69 in BC. The correlation between the expression of CCDC69 and the number of tumor-infiltrating lymphocytes was confirmed by interaction analysis of TIMER and GEPIA.

**Results:**

The results showed that CCDC69 expression was significantly lower in cancer samples than in normal tissues, and was significantly lower in highly invasive BC than in carcinoma *in situ*. Meanwhile, low levels of CCDC69 were associated with a further poor prognosis. CDCC69 expression was positively correlated with the amount of different tumor-infiltrating lymphocytes. Mechanically, it could be presumed that the low expression of CCDC69 in BC might be caused by hypermethylation of the promoter region.

**Conclusions:**

Summarily, CDCC69 could be used as a potential biomarker to predict the prognosis of BC and the sensitivity to immunotherapy such as PD-1/PD-L1 checkpoint inhibitors.

## Background

Breast cancer (BC) is one of the most common malignancies in women, threatening the physical and mental health of women all over the world (1). BC is a complex disease of morphological and molecular heterogeneity, manifested by three morphological levels and more than four different molecular subtypes, including luminal A, luminal B, HER2-positive, and basal-like BC (2). Early detection, treatment, and metastasis monitoring are very important for the prognosis of patients. Over the past decades, numerous methods have attempted to improve the diagnostic accuracy of BC, such as breast ultrasound imaging, x-ray mammography, and positioning biopsy. However, these methods often cause radioactive or invasive damage to patients, and it is difficult to distinguish the exact molecular subtypes. The emergence of biomarkers divides BC molecular characteristics into subgroups with specific disease course and homogeneous patterns that are sensitive to chemotherapy or new therapies. Diagnostic markers based on disease-specific genes and proteins contribute to the realization of precision medicine for BC. Therefore, it is very important to explore new biomarkers to help predict the clinical characteristics and prognosis of BC.

Most cancer genomic and genetic studies are conducted on heterogeneous tumor tissue samples. Studies of various tumor gene bank data provide valuable information about genomic changes in tumor samples relative to normal samples. These studies provide important tools for understanding key information about tumor initiation, progression, and metastasis. Coiled-coil domain-containing (CCDC) proteins participated in diverse functions of regulating the cell cycle and mediating apoptosis due to the highly versatile coiled-coil motif (3, 4). In recent years, studies have shown that CCDC proteins are closely related to a variety of tumors, such as lung cancer and pancreatic ductal adenocarcinoma (5, 6). CCDC69 is one of these family members, located at 5q33.1 (7). Moreover, CCDC69 was involved in controlling the assembly of the central spindle and the recruitment of intermediate region components, which was necessary for the cytoplasmic division of animal cells (7). For instance, Cui et al. confirmed that CCDC69 could enhance platinum-induced apoptosis in ovarian cancer cells and improve the sensitivity of combination therapy with platinum drugs (8). Similarly, CCDC69 overexpression activated the p14ARF/MDM2/p53 pathway in ovarian cancer, thereby improving cisplatin resistance (9). Interestingly, Li et al. also found that CCDC69 expression was negatively correlated with tumor purity in 33 TCGA tumor types (10). And CCDC69 was closely related to the overall survival of luminal BC patients and human epidermal growth factor receptor 2 (HER2)-positive BC patients (11, 12). However, up to now, little is known about the expression characteristics and mechanism of CCDC69 in breast cancer

Here, in the present study, we comprehensively evaluated the correlations between CDCC69 expression and BC clinical features and the prognosis of BC patients using public databases. Moreover, we also investigated the main pathways that CDCC69 participated in BC. The CDCC69 expression was positively correlated with the status of different tumor-infiltrating lymphocytes (TILs) and programmed death 1 (PD-1)/programmed death-ligand 1 (PD-L1) expression. Meanwhile, we investigated the potential mechanism of the low expression of CCDC69 in BC, which may be caused by hypermethylation of the promoter region. Our findings shed light on the mechanism between CDCC69 and tumor-immune interactions, posing that CDCC69 could serve as a novel and potential biomarker to predict BC prognosis and sensitivity to PD-1/PD-L1 inhibitor therapy.

## Materials and methods

### Gene expression data

RNA-Seq filings and clinical patient data, including 1,053 cancer samples and 111 normal samples were downloaded from the publicly available TCGA databases (https://www.cancer.gov/tcga). And the data about CCDC69 expression and methylation in 48 BC cell lines were retrieved from the cancer cell line encyclopedia (CCLE). No ethical conflict is needed because of no experimental data in this study.

### CCDC69 levels and survival analysis

To assess the prognostic role of CCDC69 in BC, the Kaplan-Meier plotter has been performed online (http://kmplot.com/analysis/) to determine the prognostic significance (13). The hazard ratio (HR) with 95% confidence intervals and log-rank P-value also has been computed.

### Gene enrichment analysis

The co-expression genes of CCDC69 were downloaded from the cBioPortal database and breast invasive carcinoma (TCGA, Firehose Legacy, 1,108 samples) cohort. The R packages “clusterProfiler” and “enrichplot” were used to perform the Gene Ontology (GO) project and the Kyoto Encyclopedia of Genes and Genomes (KEGG) functional pathway enrichment analyses. Meanwhile, the gene enrichment analysis (GSEA) was used to identify pathways that differed significantly in the presence of high and low CCDC69-expressing BC samples. This analysis was performed by GSEA software 3.0 from the Broad Institute. The terms in KEGG pathway with corrected P value < 0.05 and false discovery rate value < 0.25 were considered significantly enriched.

### CCDC69 expression and TILs analysis

We analyzed the correlation of CCDC69 expression with 6 types of TILs (B cells, CD4+ T cells, CD8+ T cells, neutrophils, macrophages, and dendritic cells) in BC *via* The tumor immune estimation resource (TIMER) algorithm database (https://cistrome. shinyapps.io/timer/) (14). Meanwhile, TIMER was applied to exam PD-1 and PD-L1 expression. Then, the correlation between CCDC69 expression and gene markers of TILs was detected by gene expression profiling interaction analysis 2 (GEPIA2) (15). And the lymphocyte-specific immune recruitment (LYM) metagene signature was as previously reported (16).

### Statistical analysis

The R (version 3.6.2) was used for statistical analysis (17). Wilcox test was used to analyze the CCDC69 expression difference between tumor and normal samples. The association between CCDC69 expression and the BC clinical features was analyzed by the kruskal test. The receiver operating characteristics (ROC) curve drawn by the pROC package was used to evaluate the capability of diagnosis as well as set the optimal cutoff value to identify the high CCDC69 expression group and the low CCDC69 expression group accordingly (18).

## Results

### The mRNA expression levels of CCDC69 in human cancers

**Figure 1:**
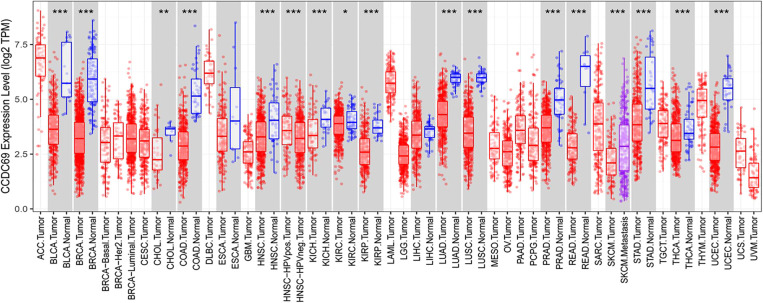
The expression level of CCDC69 in different human cancers. (**A**) The expression level of CCDC69 in different tumors in TCGA database was detected by TIMER. *P < 0.05, **P < 0.01, ***P < 0.001.

To assess mRNA expression levels of CCDC69 in human cancers, RNA-seq data derived from TCGA was analyzed by the TIMER database. This analysis revealed that the CCDC69 expression was significantly lower in bladder urothelial carcinoma (BLCA), breast invasive carcinoma (BRCA), cholangiocarcinoma (CHOL), colon adenocarcinoma (COAD), esophageal carcinoma (ESCA), head and neck cancer (HNSC), kidney chromophobe (KICH), kidney renal clear cell carcinoma (KIRC), kidney renal papillary carcinoma (KIRP), liver hepatocellular carcinoma (LIHC), lung adenocarcinoma (LUAD), lung squamous cell carcinoma (LUSC), prostate adenocarcinoma (PRAD), rectum adenocarcinoma (READ), stomach adenocarcinoma (STAD), thyroid carcinoma (THCA), and uterine corpus endometrial carcinoma (UCEC) tissues compared to the neighbor normal tissues, respectively (Figure 1).

### The correlation of CCDC69 expression with clinical features of BC

**Figure 2:**
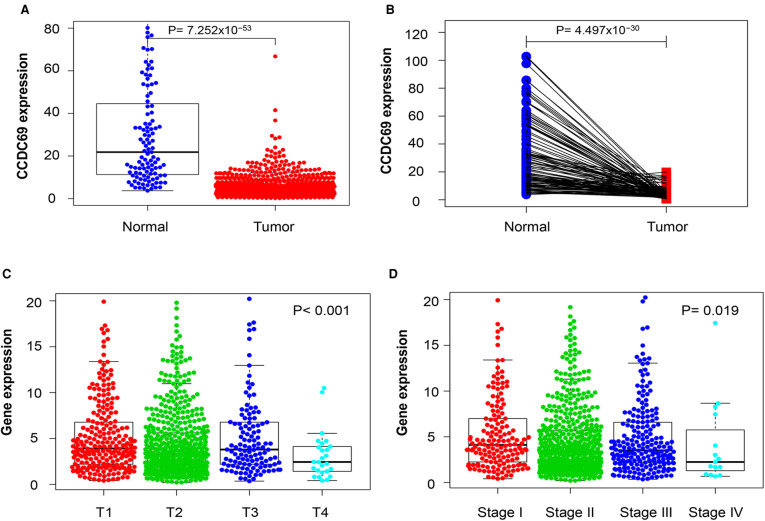
The expression level of CCDC69 was correlated with the BC clinical characteristics. (**A**) The expression level of CCDC69 in normal and BC specimens. (**B**) CCDC69 expression level of BC and paracancer tissue samples. The expression level of CCDC69 was correlated with tumor size (**C**) and pathological stage (**D**).

**Figure 3:**
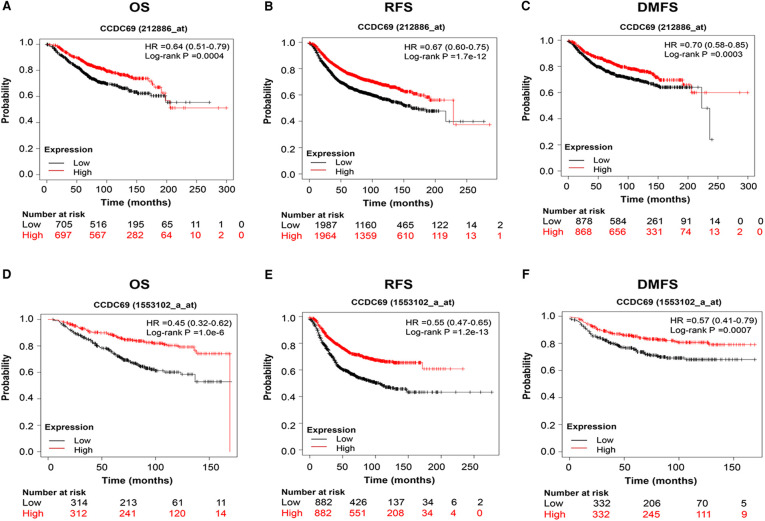
Prognostic value of CCDC69 in BC patients. (**A,D**) Overall survival curve of CCDC69 for BC patients; (**B,E**) relapse free survival curve of CCDC69 for BC patients; (**C,F**) distant metastases free survival curve of CCDC69 for BC patients.

To evaluate the relationship between CCDC69 expression levels and BC clinical features, RNA-seq data from TCGA was analyzed. As shown in Figure 2A, the expression level of CCDC69 in 1,053 BC samples was significantly lower than that in 111 normal samples (*P* = 7.252e-53). The expression of CCDC69 in 111 BC tissues was significantly reduced than in paired paracancerous samples (*P* = 4.497e-30) (Figure 2B). Meanwhile, compared with small size in BC (T1–3) and pathologic stages (Stage II-IV) in BC patients, the expression of CCDC69 was obviously below in large size (T4) and advanced pathological stage (Stage IV) patients (*P *< 0.001, *P* = 0.019) (Figures 2C,D). Moreover, the low expression of CCDC69 was found to be associated with poor overall survival (OS), relaps-free survival (RFS), and distant metatasis-free survival (DMFS) in BC based on Affymetrix microarray in the Kaplan-Meier plotter database (Figure 3). These results suggested that the expression level of CCDC69 in BC was closely related to the clinical manifestations of the patients.

### The diagnostic value of CCDC69 in BC

**Figure 4:**
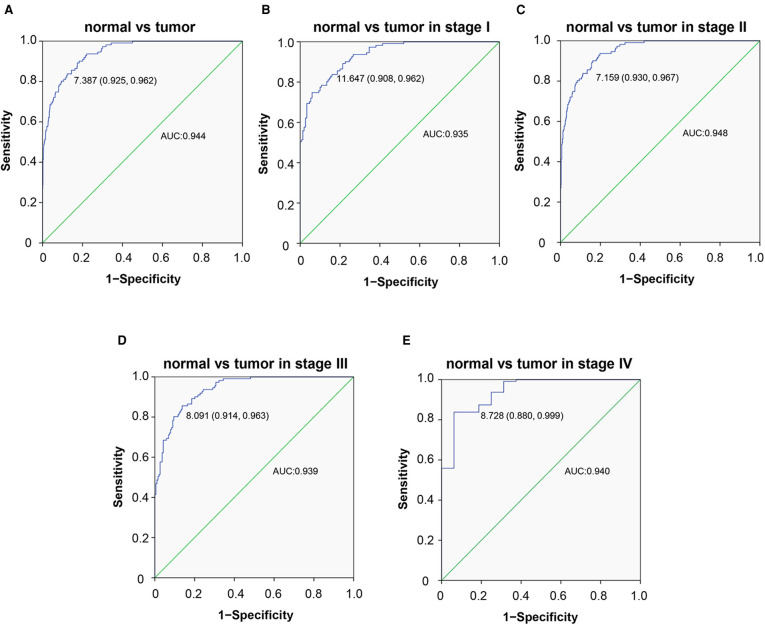
Diagnosis value of CCDC69. The ROC curve of normal tissues and BC (**A**), and subgroup analysis of stage I (**B**), II (**C**), III (**D**), and IV (**E**).

The diagnostic value of CCDC69 was further evaluated combined with the association between CCDC69 and the clinical manifestations of BC. The performance of ROC curve showed that the area under the curve (AUC) was 0.944, indicating a strong diagnostic ability and the optimal cut-off value was 7.387. In addition, by analyzing the four stages, the classification based on CCDC69 expression also showed a strong efficiency (AUC: stage I was 0.935, stage II was 0.948, stage III was 0.939, stage IV was 0.940) **(**Figures 4A–E**)**.

### The association of CCDC69 with immune response in BC

**Figure 5:**
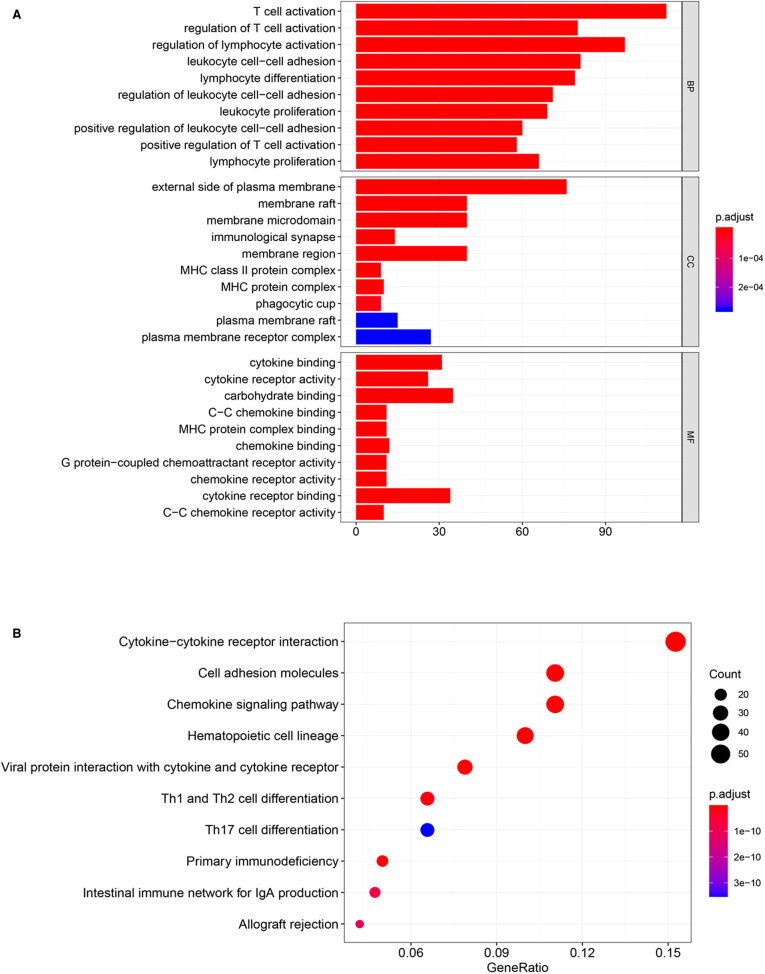
CCDC69 was involved in BC immune response. GO (**A**) and KEGG (**B**) analysis of 1,108 BC samples with CCDC69 expression levels showed that the genes positively co-expressed with CCDC69 were mainly related to immune pathways.

To explore the potential biological effect of CCDC69 in BC, we obtained the co-expression genes of CCDC69 from 1,108 samples of BC in the cBioportal database, among which 745 genes were positively correlated and no negatively correlated genes were found. Based on GO and KEGG analysis, the genes co-expressed with CCDC69 were mainly related to immune pathways, such as Cytokine-cytokine receptor interaction, Chemokine signaling pathway, Hematopoietic cell lineage, and so on (Figure 5). Furthermore, the GSEA analysis was performed on 1,053 BC samples, grouped according to the expression level of CCDC69. It was found that most of the genes in the high expression group were enriched in immune-related signaling pathways (Table 1). Taken together, the above results suggested that CCDC69 might play a biological role in BC by participating in the immune response. Therefore, it was necessary to further verify the correlation between CCDC69 and tumor immune microenvironment.

**Table 1:** GSEA indicated that CCDC69 participated in the immune response in BC.

KEGG	NES	*P-*value	FDR
Cytokine_cytokine_receptor_interaction	2.3312	0	0
Cell_adhesion_molecules_cams	2.2486	0	0
Chemokine_signaling_pathway	2.2094	0	0
Neuroactive_ligand_receptor_interaction	2.1933	0	0.0003
JAK_STAT_signaling_pathway	2.1000	0	0.0021
Hematopoietic_cell_lineage	2.0054	0.0020	0.0056
Calcium_signaling_pathway	1.9302	0	0.0108
Leukocyte_transendothelial_migration	1.9244	0.0042	0.0104
Adipocytokine_signaling_pathway	1.8800	0.0020	0.0171
Endocytosis	1.8631	0.0041	0.0190

*GSEA, gene enrichment analysis; BC, breast cancer; KEGG, Kyoto Encyclopedia of Genes and Genomes; NES, normalized enrichment score; FDR, false discovery rate*.

### The correlation between CCDC69 expression and TILs

**Figure 6:**
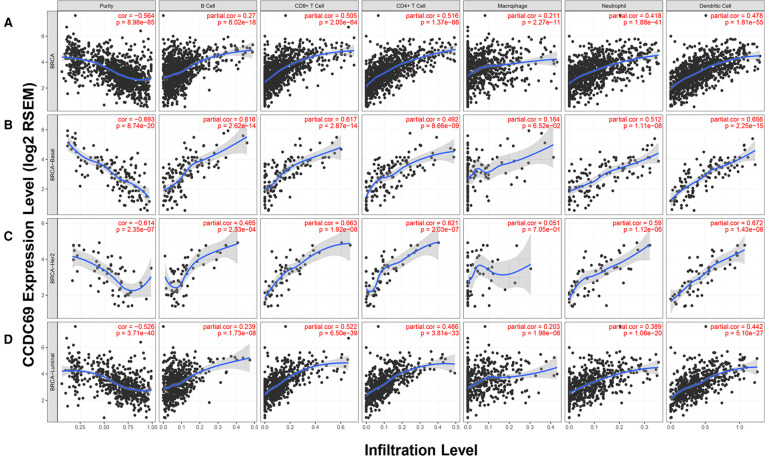
The expression of CCDC69 in the TIMER database was correlated with the infiltration level of TILs. (**A**) In BC, CCDC69 expression was significantly positively correlated with infiltration of B cells, CD8^+^ T cells, CD4^+^ T cells, macrophages, neutrophils, and dendritic cells. Analysis of TILS infiltration level and CCDC69 expression level in (**B**) basal subtype, (**C**) HER2 subtype and (**D**) luminal subtype in TIMER database.

The relationship between the infiltration of six common types of TILs and the expression level of CCDC69 was evaluated by TIMER. The results indicated that CCDC69 expression levels had a visibly positive correlation with infiltrating levels of B cells (*r* = 0.270, *P* = 8.02e-18), CD8+ T cells (*r* = 0.505, *P* = 2.05e-64), CD4+ T cells (*r* = 0.516, *P* = 1.37e-66), macrophages (*r* = 0.211, *P* = 2.27e-11), neutrophils (*r* = 0.418, *P* = 1.88e-41), and dendritic cells (*r* = 0.478, *P* = 1.81e-55) **(**Figure 6**)**. And this correlation was also consistent across different subtypes of BC (basal-like BC, luminal BC, and HER2+ BC). These results indicated that the CCDC69 expression could reflect the abundance of TILs in all anterior subtypes BC.

### The correlation between CCDC69 expression and TILs infiltration

**Figure 7:**
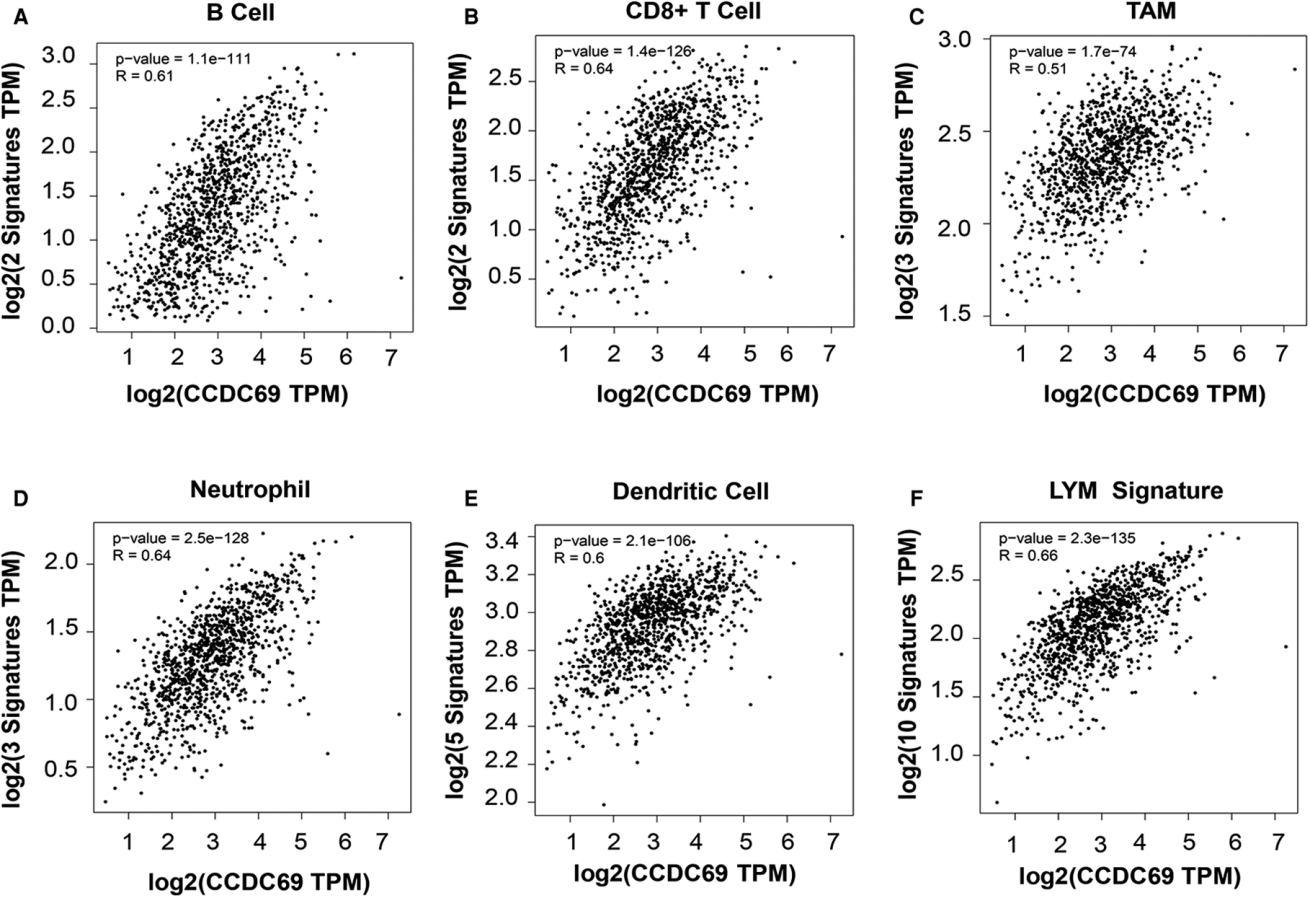
CDCC69 was positively correlated with TILs marker expression in BC. GEPIA2 was used to assess the association of CCDC69 expression levels with (**A**) CD8+ T cells, (**B**) B cells, (**C**) TAM, (**D**) neutrophils, (**E**) DC and (**F**) LYM metagenetic characteristics.

Next, the correlation between CCDC69 expression and the status of TILs marker gene expression levels in BC tissues was confirmed using the TIMER and GEPIA databases. The results showed that CCDC69 expression in BC tissues positively correlated with marker genes expression from B cell (*r* = 0.61, *P* = 1.1e-111), CD8+ cell (*r* = 0.64, *P* = 1.4e-126), TAMs (*r* = 0.51, *P* = 1.7e-74), neutrophil (*r* = 0.64, *P* = 2.5e-128) and dendritic cell (*r* = 0.6, *P* = 2.1e-106) (Figures 7A–E**)**. Meanwhile, the LYM, a signature of BC prognosis, also showed a strong positive correlation with CCDC69 (*r* = 0.66, *P* = 2.3e-135) **(**Figure 7F**)**. These results strongly suggested that CCDC69 expression reflected the level of TILs infiltration in BC.

### The correlation between CCDC69 and PD-1/PD-L1 expression levels

**Figure 8:**
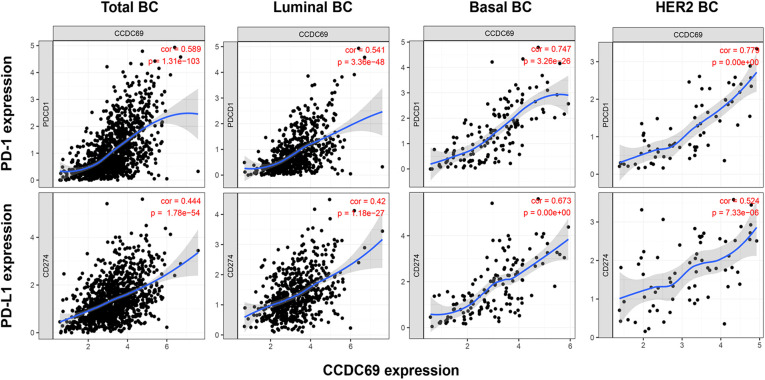
The expression levels of PD-1 and PD-L1 were decreased in BC with low expression of CDCC69. The relationship between CCDC69 expression and PD-1 and PD-L1 expression in various BC subtypes was evaluated by TIMER.

PD-1, belonging to the B7 family of immunostimulatory/inhibitory molecules, is commonly expressed on the surface of TILs (19). PD-L1 is one of the ligands for PD-1 (20). Studies have shown that the application of immune checkpoint inhibitors targeting the PD1/PD-L1 axis could observably improve the prognosis of cancer patients (21). Furthermore, the anti-PD-1 antibody atezolizumab has been approved by the U.S. Food and Drug Administration for use in combination with nab-paclitaxel for the treatment of metastatic TNBC (22, 23). Based on these theories, the correlation between CCDC69 and PD-1/PD-L1 axis was explored by TIMER, which showed that the expression level of CCDC69 was positively correlated with the expression of PD-1 and PD-L1 in all subtypes of BC **(**Figure 8**)**. Therefore, CCDC69 could reflect the expression of immune checkpoints to some extent, and patients with low CCDC69 expression might have a lower response rate to PD-1 and PD-L1 inhibitors.

### The methylation level in the promoter region affected CCDC69 expression

**Figure 9:**
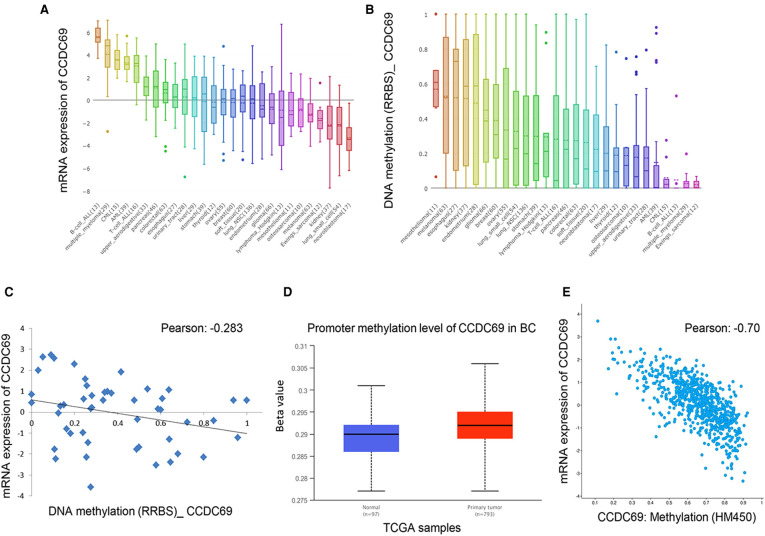
The expression level of CCDC69 was affected by the methylation level of its promoter region. (**A**) The expression of CCDC69 in various cancer cell lines. (**B**) Levels of DNA methylation in various cancer cell lines. (**C**) DNA methylation was negatively correlated with CCDC69 expression in 48 BC cell lines. (**D**) In the UALCAN database, the promoter methylation level of the breast cancer sample CCDC69 was higher than that of normal tissues. (**E**) Analysis of Bioportal data indicated that the expression level of CCDC69 was negatively correlated with the methylation level of the promoter region.

The CCDC69 expression was relatively highly expressed in B-cell_ALL, multiple myeloma, CML, AML, T-cell_ALL, upper aerodigestive, pancreas, colorectal, and esophagus. On the contrary, the CCDC69 expression was relatively low expressed in the endometrium, glioma, lymphoma Hodgkin, mesothelioma, osteosarcoma, melanoma, Ewings sarcoma, kidney, lung small cell, neuroblastoma (Figure 9A). We also analyzed the levels of DNA methylation in each cell line. Compared with CCDC69 expression, DNA methylation was an important factor affecting CCDC69 expression. More specifically, the higher the degree of methylation, indicated the lower the expression of CCDC69 (Figure 9B). Spearman's Correlation analysis of 48 BC cells showed that DNA methylation was negatively correlated with the expression level of CCDC69 (Figure 9C). According to the UALCAN database, the promoter methylation level of CCDC69 in BC samples was higher than that in normal tissues (Figure 9D). Moreover, the cBioPortal data indicated that the CCDC69 expression level was negatively correlated with the promoter region methylation level (Figure 9E). Taken together, these results suggested that the CCDC69 low expression in BC may be possibly caused by hypermethylation of the promoter region.

## Discussion

This study supported that CCDC69 served as a prognostic biomarker in BC. CCDC69 mRNA levels were obviously lower in invasive breast carcinoma tissues compared with adjacent normal tissues. CCDC69 mRNA levels were inversely correlated with tumor size, pathological stage of BC patients. Furthermore, CCDC69 expression correlated with the numbers of TILs based on the levels of markers for different immune cell types in BC. Thus, our study puts insights into understanding the potential role of CCDC69 in tumor immunology and its potential as a BC biomarker.

At present, some preliminary studies on CCDC69 in BC suggest that CCDC69 may be associated with overall survival in BC patients (11, 12). But these studies have some limitations. Firstly, in the above reports, the studies on the relationship between CCDC69 and patient prognosis mainly focused on luminal and HER2+ BC, which did not have the generality of all BC subtypes. Then, only the correlation between CCDC69 and overall survival was investigated without delving into the potential biological function. Thus, there is a need to further explore the role of CCDC69 in BC. In our study, we investigated the potential biological functions and mechanisms of CCDC69. CCDC69 was decreased in BC and 16 other types of cancer tissues than in corresponding normal tissues. And the low expression level of CCDC69 in advanced stages suggested that the expression level of CCDC69 was reduced in invasive disease. Moreover, we found that in BC cell lines, the expression level of CCDC69 was inversely proportional to the methylation level, suggesting that the hypermethylation of the CCDC69 promoter may lead to the reduction of mRNA level.

Using GSEA analysis, we found that CCDC69 was involved in immune response in BC, and this was a previously unreported function. In the past few years, immunotherapy has been identified as an important cancer treatment (24–26), with remarkable activity and therapeutic potential for combating multiple tumor types (27). BC with different degrees of TILs infiltration has different efficacy in chemotherapy, radiotherapy, or immunotherapy. For example, high TILs infiltration was associated with a lower risk of recurrence in BC patients (28, 29). In addition, the assessment of immune environment regulation after neoadjuvant chemotherapy in BC patients showed that in some patients, the infiltration of immune microenvironment TILs was significantly increased (30, 31). These results suggested that risk stratification with appropriate immune markers could be used to effectively determine the risk of recurrence in early disease setting. Here, we evaluated the relationship between CCDC69 and TILs in BC. In all BC subtypes, the expression of CCDC69 was positively correlated with TILs indicating that CCDC69 could reflect the infiltration of TILs.

In addition, the same results were found in CCDC69 and PD-L1/PD-1. With the application of PD-1/PD-L1 inhibitor therapy, the biomarkers to predict BC's reaction to ICB agents also deserve attention. With the application of PD-1/PD-L1 antibodies therapy, biomarkers that predict the drug response of BC to PD-1/PD-L1 antibodies also deserve attention. Moreover, the PD-L1 expression detected by immunohistochemical has been proved to be an effective biomarker for selecting patients who benefit from anti-PD-1 and anti-PD-L1 antibodies (32, 33). The results showed that CCDC69 was positively correlated with PD-L1/PD-1 expression, suggesting that CCDC69 could be considered as a predictor of the benefit of anti-PD-1/anti-PD-L1 antibody treatment in BC patients.

In order to avoid the bias of experimental conclusions, we adopted the method of combining multiple databases and multiple analyses, which supported our conclusion from the clinical characteristics of the tumor to the potential molecular mechanism. Admittedly, there are some limitations to our study. For example, we should collect information on BC patients diagnosed in the last 10 years or longer, which can be used to verify the relationship between survival and CCDC69. Regrettably, due to some difficulties in sample resources and collection, we did not collect appropriate clinical samples for verification. Further, hematoxylin and eosin staining and immunohistochemistry can be used to determine the type of TILs in later studies. Moreover, due to the limitation of bioinformatics technology and data in the database, there has not been a comparative analysis of CCDC69 expression levels in various breast cancer subtypes in this study. Lastly, this study mainly discussed the role of CCDC69. Although single-gene diagnosis is simple and convenient, its accuracy and sensitivity are not as good as multi-gene diagnosis. Therefore, subsequent study verification is required which will make our conclusion more credible. Totally, CCDC69 expression is expected not only to be a diagnostic and prognostic marker, but also to help predict the drug response of patients to immunotherapy.

## Conclusions

Together, our study demonstrated that the CCDC69 expression was closely related to BC clinical features and the low expression level of CCDC69 was associated with an unfavorable prognosis. Importantly, CCDC69 expression was positively connected to TILs quantities and PD-L1/PD-1 expression. CCDC69 might be a potential biomarker to help identify BC patients who would benefit more from anti-PD-L1/anti-PD-1 antibodies.

## Data Availability

The datasets presented in this study can be found in online repositories. The names of the repository/repositories and accession number(s) can be found in the article/Suplementary Material.
